# Exploring paruresis (‘shy bladder syndrome’) and factors that may contribute to it: a cross-sectional UK survey study

**DOI:** 10.1136/bmjopen-2024-086097

**Published:** 2024-11-17

**Authors:** Hayley Anne Hutchings, Abi Kehinde

**Affiliations:** 1 School of Medicine, Swansea University, Swansea, UK

**Keywords:** Patient Reported Outcome Measures, Risk Factors, Surveys and Questionnaires, Bladder disorders

## Abstract

**Abstract:**

**Objectives:**

To assess the prevalence and severity of paruresis (‘shy bladder syndrome’) in a population of university staff and students and to determine if there was any relationship between demographics, self-esteem, presence of social anxiety disorders and negative toilet experiences and paruresis.

**Design:**

We undertook an anonymised cross-sectional online survey using Microsoft Forms. We invited participants aged 18 and over to complete the survey which included demographic information; any pre-existing medically or self-diagnosed anxiety-related conditions; Shy Bladder Scale (SBS); Rosenberg Self-Esteem Scale (RSES) and questions about using school toilets in their younger life. We defined ‘mild’ and ‘severe’ paruresis based on total SBS cut-off scores of greater than or equal to 31 and greater than or equal to 40. We calculated prevalence of paruresis, and explored differences in self-esteem, school toilet experience and social anxiety disorders between individuals with and without paruresis. Multivariable logistic regression was used to determine which variables had any influence on having a ‘mild’ and ‘severe’ paruresis diagnosis.

**Setting:**

We distributed the survey to all staff and students via their university email address as well as promoting the survey on university social media sites.

**Results:**

We received responses from 356 individuals. Most participants (237, 66.6%) were within the 18–30 year age category and most (277, 77.8%) were white. There were 221 (62.1%) females, 119 (33.4%) males and 16 (4.5%) other genders. The prevalence of ‘mild’ paruresis was 25.8% and of ‘severe’ paruresis 14.9% in this sample. 73.0% indicated that they had at least one medically or self-diagnosed anxiety disorder. There was a statistically significant difference in the total SBS score between individuals with and without an existing anxiety disorder (8 vs 19, <0.001). The adjusted odds of ‘mild’ paruresis were higher for men than women (OR 3.39; 95% CI 1.90 to 6.06), lower for those with a lower RSES score (OR 0.90; 95% CI 0.85 to 0.96), lower for those for a lower school toilet experience score (OR 0.88; 95% CI 0.78 to 0.99) and higher for those with at least one anxiety disorder (OR 3.164; 95% CI 1.52 to 6.59). The adjusted odds of ‘severe’ paruresis were higher for men than women (OR 2.60; 95% CI 1.32 to 5.12), lower for those with a lower RSES score (OR 0.90; CI 0.84 to 0.97) and higher for those with at least one anxiety disorder (OR 2.99 (1.18–7.56)).

**Conclusion:**

Large organisations should consider measures to help manage anxiety and improve toilet experiences. These could include resources and signposting to manage anxiety disorders and single-occupancy toilets and toilets in quiet areas to limit anxiety associated with shy bladder syndrome.

**STRENGTHS AND LIMITATIONS OF THIS STUDY**

- We used a broad range of validated questionnaires to explore relationships between social anxiety disorders, self-esteem, poor experiences in school toilets and the prevalence and severity of paruresis.
- We undertook an anonymised survey with university staff and students that included all genders and a wide age range.
- There was no medical assessment involved and all paruresis and anxiety symptoms were based on self-reports.
- The experience of using school toilet questionnaire may be subject to recall bias for older individuals who may not remember their experiences.
- The sample may be motivated and may not be generalisable to the wider population.

## Introduction

 Paruresis refers to the inability to initiate or sustain effective urination (micturition) in situations where there is a perception of scrutiny, or potential scrutiny, by others.^1^ It normally manifests in public toilets and as a result, individuals generally have difficulty or inability to urinate. It is often referred to as ‘shy bladder syndrome’. The reasons for the difficulties and/or inhibition of micturition include busy toilets with an increased number of people present; being close to other individuals and the type of toilet.^1^,^3^ Paruresis was first described in 1954 by Williams and Degenhardt^4^ and is thought to be a function of a psychosomatic disorder and a phobia of urination.^5 6^ It is known to cause distress and can impact on social activities, relationships, work activities and quality of life.^6^,^9^ The severity of paruresis can range from slight hesitancy of using public toilets to chronic urinary retention.^10^

The prevalence of paruresis is debatable. The International Paruresis Association (IPA) (USA and Canada) and the UK Paruresis Trust (UKPT) suggest that the prevalence of paruresis is around 7%,^11 12^ but a systematic review conducted in 2017 suggested that the range may be somewhere between 2.8% and 16.4% for mixed sex samples^9 13^ and as much as 32% in male only samples.^3 13 14^ The IPA and the UKPT suggest that people of any sex can experience paruresis,^11 12^ but other research studies suggest that males are more likely than females to have paruresis.^15^,^17^ The age of onset of paruresis is also considered debatable.^11 12^ It has been linked with adolescence.^3^ Some paruresis studies, however, have documented that the mean age of those experiencing paruresis is between 29 and 41 years, but data are limited.^16^,^18^ There is currently no information regarding the ethnicity of those with reported symptoms.^9^

The precise triggers for paruresis are uncertain,^16^ but there are some suggestions of it occurring as a result of anxiety or other psychosomatic problems, distress or personal conflicts.^1 19 20^ Some research studies have demonstrated the links between individuals suffering with paruresis and having other social anxiety issues. These include panic symptoms, generalised anxiety disorders and stuttering;^4 13 17^ as well as problems with alcohol dependence, depression and obsessive compulsive disorder (OCD).^6^ Extroversion has been linked to increased well-being,^21 22^ with claims that the association is substantial.^23^ Experimental research exploring this concept has however been limited,^22^ and possible links between introversion/extroversion and paruresis have not been previously examined.

Research exploring standards and behaviours in school toilets have highlighted issues such as poor cleanliness, inadequate resources and washing facilities and bullying which could dissuade pupils from using school toilets.^24^,^26^ Anecdotal evidence from the UKPT suggests that many individuals attending their workshops to manage paruresis indicate that they first experienced problems when they used toilets away from home in their childhood, with their earliest memories of difficulties relating to their school days.^27^ Avoidance of school toilets may result in future anxiety when using public toilets and this could initiate paruresis. Unlike other social anxiety disorders, such as OCD, the limited research to date suggests that it does not appear to be linked to contamination/cleanliness of the public toilets.^6^

Given the limited literature focusing on paruresis, the purpose of this study was to explore: (1) the prevalence of ‘mild’ and ‘severe’ paruresis; (2) whether there was any relationship between existing social anxiety disorders and the presence and severity of paruresis; (3) whether self-esteem was linked to paruresis and (4) whether negative experiences of using school public toilets had any influence on the development of paruresis.

## Methods

We created a survey using Microsoft Forms and invited participants over the age of 18 to take part. Staff and students from Swansea University were sent an email which summarised the study and which attached a detailed participant information sheet. The total staff and student numbers at Swansea University are approximately 3300 and 20 500, respectively. The link to take part in the study was included in the email. We also advertised the study on relevant university social media sites via snowballing methods. The survey was open from 1 November 2023 until 31 January 2024. No personal data were collected, and aggregate categories were used (where appropriate) to avoid identification. Only limited demographic information was collected based on previously published literature identifying factors that may contribute to paruresis development.^9 11 12 15 17^

Following completion of the consent to participate questions, participants completed five survey sections:

- Section 1: general demographic information (age, ethnicity and gender). These variables were all collected as categorical data.
- Section 2: existing anxiety disorders and if yes if they were self-diagnosed or medically diagnosed.
- Section 3: Shy Bladder Scale (SBS).^15^ This was composed of 19 questions, with five response options (0=very little; 1=a little; 2=some; 3=much and 4=very much).
- Section 4: Rosenberg Self-Esteem Scale (RSES).^28 29^ This was composed of 10 questions, with four response options (strongly agree; agree; disagree and strongly disagree).
- Section 5: questions relating to experiences of using school toilets. This was composed of seven questions with four response options (cannot remember; never; sometimes and always). Based on the works of Barnes and Maddocks.^24^

Responses to the questions in sections 3, 4 and 5 were mandatory and participants could not proceed until a response was given to each question. This ensured complete data collection for the key questionnaires.

### Data management

The information from the MS Forms survey was exported into SPSS V.29 (SPSS, Chicago, IL, USA) for analysis.

The SBS^15^ and the RSES were scored according to the developers’ guidance.^28 29^ The survey was set up such that individual question responses to the SBS, RSES and school toilet experience questionnaires were mandatory. This ensured that there were no missing data for these questionnaires and as such we did not need to undertake any imputation of the data.

We scored three SBS subscale scores (Difficulty urinating in public; Interference and distress and Fear of negative evaluation) as well as a total SBS score. The difficulty urinating in public subscale was made up of seven questions (Q1, 3, 4, 6, 7, 12 and 13). Interference of distress subscale was made up of six questions (Q8, 15, 16, 17, 18 and 19). The fear of negative evaluation subscale was made up of six questions (Q2, 5, 9, 10, 11 and 14). The total SBS score was calculated using the responses from all 19 questions. We scored the Shy Bladder subscale and total scores by totalling the individual question responses from 0 to 4. The total score ranged between 0 and 76; the difficulty urinating in public subscale ranged from 0 to 28; the interference of distress subscale ranged from 0 to 24 and the fear of negative evaluation subscale ranged from 0 to 24. A higher score indicated worse paruresis.

Previous research developing the SBS documented that the lowest SBS score obtained by a participant with clinically diagnosed paruresis was 31. The sensitivity and specificity of this score against the self-rated classification of paruresis using the Mini International Neuropsychiatric Interview for DSM-IV^30^ were 1.0 and 0.89, respectively.^15^ We therefore used the threshold value of 31 to define ‘mild’ paruresis. A total SBS score of 40 or more demonstrated the most favourable combination of sensitivity (true positives, 0.96) and specificity (true negatives, 0.96) in the SBS development study.^15^ We therefore used the threshold value of 40 or more to define ‘severe’ paruresis.^15^

We scored the RSES by totalling the individual four-point items after reverse-scoring the negatively worded items. The score ranges from 0 to 30, where a score of 15–25 is average, and a score below 15 may suggest low self-esteem.^28 29^

We calculated a total score for the school toilet experience questionnaire. For positively worded questions, we scored a response of ‘cannot remember’ as 0; ‘never’ as 1; ‘sometimes’ as 2 and always as 3. For negatively worded questions, we scored a response of ‘never’ as 3; ‘sometimes’ as 2 and always as 1. We calculated a total school toilet experience score by adding the responses to each of the seven questions to give a score between 7 and 21. A higher score indicated better experience of using school toilets. As this questionnaire was adapted from a previous questionnaire which has no defined cut-offs, we defined a ‘negative’ school toilet experience score based on individuals scoring negatively to more than 50% of questions. We classified scores of 10 or less as having a ‘negative experience’ and scores of 11 or more as having an ‘adequate/positive experience’.

### Statistical analysis

We conducted statistical analyses using SPSS V.29 (SPSS, Chicago, IL, USA). Demographic information are presented as numbers and percentages. We visually examined the distribution of scores for the SBS total and SBS subscale scores, RSES and school toilet experience for normality. Visual inspection suggested that the data were not normally distributed. We further confirmed this using the Kolmogorov-Smirnov and the Shapiro-Wilk tests,^31^ which identified that all p values were less than 0.05 and that all scores were statistically significantly different from normal. We therefore present these data as medians (lower quartile and upper quartile). We calculated the prevalence of ‘mild’ paruresis in our sample by dividing the number of people in the sample with a SBS score of greater than or equal to 31 by the total population size; and the prevalence of ‘severe’ paruresis by dividing the number of people in the sample with a SBS of greater than or equal to 40 by the total population size.^15^ We explored SBS scores in those individuals with one or more social anxiety disorder compared with those with no social anxiety disorders using the non-parametric Mann-Whitney U test where the presence of one or more social anxiety disorder was compared with having no social anxiety disorders. We used non-parametric Spearman correlation to examine the relationship between RSES and SBS total and subscale scores and the relationship between experience of using school toilets and SBS scores. We used a p value of p<0.05 to indicate statistically significant results. Based on good practice for reporting on statistically significant results, p values larger than 0.01 were reported to two decimal places, those between 0.01 and 0.001 to three decimal places; p values smaller than 0.001 were reported as p<0.001.^32^

Finally, we used multivariable logistic regression analysis to determine whether any of the variables included in the model had any influence on having a ‘mild’ and ‘severe’ paruresis diagnosis (Y) or not (N) (where Y was defined as having a SBS total score of 31 or more; or 40 or more). The selection of independent variables was initially based on existing literature proposing links between paruresis and gender, self-esteem and pre-existing anxiety disorders. We further explored relationships between paruresis and self-esteem and school toilet experience using bivariate correlation (SBS vs RSES, and SBS vs School Toilet Experience). Only those correlations that were statistically significant were added to the logistic regression model.

Age, gender, medically or self-diagnosed anxiety disorders were classified as categorical independent variables and school experience score, and RSES were classified as the continuous independent variables. We assessed goodness of fit using the Omnibus test of Model Coefficients and the Hosmer and Lemeshow test.

### Sample size

We calculated our sample size based on recent guidelines for adequate sample sizes for logistic regression.^33^ Using the formula n=100+50 i (where i represents the number of independent variables in the final model), based on five variables, we calculated that we needed a sample size of at least 350 to detect a medium to large effect size.

### Public and patient involvement

It was not appropriate or possible to involve patients or the public in the design, or conduct, or reporting or dissemination plans of our research.

## Results

We received responses from 356 participants. Most participants were aged between 18 and 30 years (237/356, 66.6%), were female (221/356, 62.1%) and were white (277/356, 77.8%). Table 1 illustrates the demographic characteristics of the responding participants, the proportion experiencing ‘mild’ and ‘severe’ paruresis, low self-esteem and a negative school toilet experience. Median (lower quartile, upper quartile) scores for the SBS, RSES and school toilet experience are also presented.

**Table 1:** Characteristics of the study participants (n=356 for all variables)

Variable	Frequency, N (%)
Age	
18–30	237 (66.6)
31–40	51 (14.3)
41–50	34 (9.6)
51–60	27 (7.6)
61–70	6 (1.7)
Over 70	1 (0.3)
Ethnicity	
White	277 (77.8)
Mixed	37 (10.4)
Asian	24 (6.7)
Black	15 (4.2)
Other	3 (0.8)
Gender	
Female	221 (62.1)
Male	119 (33.4)
Other	16 (4.5)
Anxiety disorders (AD)	
No AD	96 (27.0)
One or more AD	260 (73.0)
‘Mild’ SBS	
No (SBS total score less than 31)	264 (74.2)
Yes (SBS total score 31 or more)	92 (25.8)
‘Severe’ SBS	
No (SBS total score less than 40)	303 (85.1)
Yes (SBS total score 40 or more)	53 (14.9)
RSES	
Low self-esteem (RSES less than 15)	154 (43.4)
Normal self-esteem (RSES more than 15)	202 (56.6)
School toilet experience	
Positive toilet experience (score of 11 or more)	336 (94.4)
Negative toilet experience score of 10 or less)	20 (5.6)
	Median (lower quartile, upper quartile)
SBS total	15 (6, 31)
SBS difficulty urinating	5 (1, 15)
SBS interference	0 (0, 3)
SBS negative evaluation	8 (4, 13)
RSES	16 (13, 19)
School toilet experience	14 (13, 16)

RSESRosenberg Self-Esteem ScaleSBSShy Bladder Scale

A high proportion of participants were experiencing some level of paruresis with 92/356 (25.8%) exhibiting ‘mild’ paruresis (based on a total SBS score of 31 or more) and 53/356 (14.9%) exhibiting ‘severe’ paruresis (based on a total SBS score of 40 or more). A large number of participants were also experiencing low self-esteem (154/356 (43.4%), based on a RSES score of less than 15). The school toilet experience scores indicated that most participants (336/356 (94.4%)) had an adequate/positive experience of school toilets in school.

Online supplemental figure S1Online supplemental figure S1 illustrates the distribution of total SBS, RSES and school toilet experience scores across the sample of respondents. The highest total SBS score was 71 out of a possible maximum score of 74. This illustrates that many participants were experiencing a large number of paruresis symptoms for most of the time. Similarly, scores for the RSES exhibited a large range indicating that a high proportion of respondents were exhibiting low self-esteem.

Online supplemental table S1 illustrates the frequency of reported anxiety disorders in the survey participants. There were high prevalences of self-reported and medically diagnosed anxiety disorders. General anxiety disorder (GAD), social anxiety disorder and phobia were reported most frequently at 173/356 (48.6%), 127/356 (35.7%) and 106/356 (29.8%), respectively. The other anxiety disorders were also frequently reported with at least 13% of respondents reporting these disorders.

Table 2 illustrates the median (lower quartile and upper quartile) scores for the SBS total and subscale scores, RSES and school toilet experience scores split between those participants classified as having ‘mild’ or ‘severe’ paruresis or not. Non-parametric Mann-Whitney U tests identified that there was a statistically significant differences in the RSES scores (p<0.05) between those participants classified as having ‘mild’ or ‘severe’ paruresis and those classified as not having paruresis. There was also a statistically significant difference in school toilet experience scores between those participants classified as having ‘mild’ paruresis and not. There was no statistically significant difference between school toilet experience scores in those classified with ‘severe’ paruresis and not.

**Table 2:** Median (lower quartile, upper quartile) scores for the SBS, RSES and school toilet experience scales, split by participants defined as having ‘mild’ paruresis (SBS total score of 31 or more) or not; and ‘severe’ paruresis (SBS total score of 40 or more) or not

Variable	Median (lower quartile, upper quartile)		P value
	No paruresis (SBS total score of less than 31) n=264	Paruresis (SBS total score of 31 or more) n=92	
Shy Bladder Scale (SBS) score	10 (3.25, 18)	41.5 (35, 51.75)	
Shy Bladder Difficulty Urinating score	2 (0, 7)	20 (16, 24)	
Shy Bladder Interference score	0 (0, 0)	7.5 (3, 13)	
Shy Bladder negative evaluation score	6 (2, 10)	15.5 (11, 20)	
Rosenberg Self-Esteem Scale (RSES) score	16 (13, 19)	14 (11.25, 17)	<0.001*
School toilet experience score	15 (13, 16)	14 (12, 15)	0.005*
	No paruresis (SBS total score of less than 40) n=303	Paruresis (SBS total score of greater than or equal to 40) n=53)	
Shy Bladder Scale (SBS) score	12 (4, 23)	49 (44, 56)	
Shy Bladder Difficulty Urinating score	4 (0, 10)	23 (19, 25)	
Shy Bladder Interference score	0 (0, 1)	12 (12, 15)	
Shy Bladder negative evaluation score	7 (3, 12)	18 (12, 21)	
Rosenberg Self-Esteem Scale (RSES) score	16 (13, 19)	14 (10, 17)	0.003*
School toilet experience score	14 (13, 16)	14 (12, 15)	0.06

*P<0.05.

Table 3 illustrates the median (lower quartile and upper quartile) scores for the SBS total and subscale scores, RSES scores and school toilet experience scores split between those participants who reported at least one self or medically diagnosed anxiety disorder compared with those participants who reported no anxiety disorder. All SBS scores and RSES scores showed a statistically significant difference between those participants who reported a self or medically diagnosed anxiety disorder compared with those participants who reported no anxiety conditions. Scores showed evidence of worse paruresis and lower self-esteem in those with an anxiety condition. There was no statistically significant difference between groups with respect to school toilet experience scores.

**Table 3:** Median (lower quartile and upper quartile) scores for the SBS, RSES and school toilet experience scales, split by participants who indicated at least one self or medically diagnosed anxiety disorder or not

Variable	Median (lower quartile, upper quartile)		P value
	No anxiety disorder (n=96)	At least one anxiety disorder (n=260)	
Total Shy Bladder Scale (SBS) score	8 (2.25, 16.75)	19 (8.25, 34)	<0.001*
Shy Bladder Difficulty Urinating score	2 (0, 10)	7 (1, 17)	<0.001*
Shy Bladder Interference score	0 (0, 0)	0 (0, 4)	<0.001*
Shy Bladder negative evaluation score	4.5 (1, 8)	9.5 (5, 15)	<0.001*
Rosenberg Self-Esteem Scale (RSES) score	19 (14.25, 22)	14.5 (12, 18)	<0.001*
School toilet experience score	14 (12, 16)	14 (13, 16)	0.385

*P<0.05.

RSESRosenberg Self-Esteem ScaleSBSShy Bladder Scale

Table 4 illustrates Spearman correlation analysis exploring the relationship between paruresis and self-esteem and paruresis and school toilet experience. Based on the statistically significant correlations between the total SBS and the RSES and the total SBS and the school toilet experience scores, the RSES and school experience scores were confirmed as candidates for the regression model.

**Table 4:** Non-parametric Spearman correlation analysis to explore the relationship between paruresis (SBS scores), self-esteem (RSES) and previous toilet experience (school toilet experience score)

SBS scores	RSES	School toilet experience score
Total Shy Bladder Scale (SBS) score	r=−0.30p<0.001*	r=−0.12p=0.02*
Shy Bladder Difficulty Urinating score	r=−0.20p<0.001*	r=−0.11p=0.04*
Shy Bladder Interference score	r=−0.18p<0.001*	r=−0.18p<0.001*
Shy Bladder negative evaluation score	r=−0.43p<0.001*	r=−0.09p=0.10

*P<0.05.

RSESRosenberg Self-Esteem ScaleSBSShy Bladder Scale

Table 5 illustrates the variables that had a statistically significant effect on whether a participant had self-reported (a) ‘mild’ or (b) ‘severe’ paruresis. Online supplemental table S2 illustrates that the Omnibus test of Model Coefficients p values were less than 0.05 and that and Hosmer and Lemeshow test p values were greater than 0.05 indicating that the model fit was appropriate for both models.

**Table 5:** Binary logistic regression data illustrating the predictor (independent) variables that had a statistically significant effect on the having (A) mild paruresis (SBS total score greater than or equal to 31); or (B) severe paruresis (SBS score greater than or equal to 40)

Variable		OR	95% CI		P value
(A) mild paruresis (SBS total score greater than or equal to 31)					
Age	18–30	1			
	31–40	1.021	0.467	2.236	0.958
	41–50	0.694	0.257	1.873	0.471
	51–60	1.230	0.425	3.558	0.702
	Over 60	2.792	0.526	14.829	0.228
Gender	Female	1			
	Male	3.392	1.900	6.056	<0.001*
	Other	1.287	0.406	4.077	0.668
RSES		0.903	0.849	0.961	0.001*
School toilet experience		0.882	0.781	0.996	0.042*
Self or medically diagnosed anxiety disorder		3.164	1.519	6.593	0.002*
(B) Severe paruresis (SBS score greater than or equal to 40)					
Age	18–30	1			
	31–40	1.086	0.413	2.859	0.867
	41–50	1.066	0.334	3.400	0.914
	51–60	2.456	0.771	7.823	0.129
	Over 60	3.687	0.598	22.743	0.160
Gender	Female	1			
	Male	2.601	1.321	5.122	<0.006*
	Other	1.947	0.560	6.768	0.294
RSES		0.902	0.837	0.973	0.007*
School toilet experience		0.941	0.812	1.086	0.407
Self or medically diagnosed anxiety disorder		2.987	1.179	7.564	0.021*

* P<0.05.

RSESRosenberg Self-Esteem ScaleSBSShy Bladder Scale

The adjusted odds of ‘mild’ paruresis were higher for men than women (OR 3.39; 95% CI 1.90 to 6.06), lower for those with a lower RSES score (OR 0.90; 95% CI 0.85 to 0.96), lower for those for a lower school toilet experience score (OR 0.88; 95% CI 0.78 to 0.99) and higher for those with at least one anxiety disorder (OR 3.164; 95% CI 1.52 to 6.59).

The adjusted odds of ‘severe’ paruresis were higher for men than women (OR 2.60; 95% CI 1.32 to 5.12), lower for those with a lower RSES score (OR 0.90; CI 0.84 to 0.97) and higher for those with at least one anxiety disorder (OR 2.99 (1.18–7.56).

## Discussion

In this cross-sectional survey, we found a high prevalence of both self-reported ‘mild’ and ‘severe’ paruresis (‘shy bladder syndrome’) of over 25% and almost 15%, respectively. A high proportion of participants were also experiencing at least one medically or self-diagnosed anxiety disorder (73%) and over half of the sample exhibited low self-esteem (56.7%). Having a social anxiety disorder resulted in a worse severity of paruresis symptoms. Those participants with an anxiety disorder had a significantly higher total SBS score than those participants without an anxiety disorder. We found that low self-esteem was associated with worse paruresis symptoms, but that there was only a weak correlation between poor toilet experiences in school and paruresis. Being male, having a medically or self-diagnosed anxiety disorder, low self-esteem and having a negative school toilet experience increased the odds of having ‘mild’ paruresis. Being male, having a medically or self-diagnosed anxiety disorder and low self-esteem increased the odds of having ‘severe’ paruresis.

We surveyed staff and students at a large university as well as promoting the study via university social media channels. The sample could therefore have been much higher, and the respondents may represent a self-motivated group who responded because they were experiencing shy bladder problems. Our reported prevalence of ‘severe’ paruresis of almost 15% was at the higher range of reported rates of between 2.8% and 16% for mixed samples identified in other studies.^3 9 13 14^ Our sample was made up of 66% females, which may account for the lower prevalence compared with other studies that have documented rates as high as 32% in only male samples.^3 13 14^ We identified a higher prevalence of ‘mild’ paruresis, which indicates that a high proportion (over 25%) of respondents were experiencing at least some problems with urination, which are likely to impact on their day-to-day activities and quality of life. In our study, we found that males were more likely to experience paruresis, which concurs with previous studies.^15^,^17^

Age was not found to be a predictor for ‘mild’ or ‘severe’ paruresis. Previous studies have documented that the mean age of those experiencing paruresis is between 29 and 41.^16^,^18^ Our sample was, however, drawn from a university sample with most participants being in the 18–30 year age category. In addition, as we only collected age category rather than actual age, it is difficult to compare the age information with other studies. Further research with a more diverse sample is needed to determine whether age is a factor for a paruresis diagnosis.

Limited information is available regarding links between ethnicity and paruresis.^9^ Our sample was predominantly white (almost 78%), so further work is needed with a more diverse ethnic sample to confirm whether or not ethnicity has any influence. As with other previous research, we found that a large proportion of our sample had at least one medically or self-diagnosed anxiety disorder. Almost 50% of our participants experienced GAD, 36% had social anxiety and almost 30% exhibited phobia. This concurs with previous studies that identified the coexistence of these disorders in individuals with paruresis.^4 6 13 17^

Although extroversion has been previously linked to increased well-being,^21^,^23^ we are not aware of any research to date that has explored links between extroversion and paruresis. Our findings indicated that low self-esteem was linked with both ‘mild’ and ‘severe’ paruresis. Poor experience of using school toilets was significant for predicting ‘mild’ paruresis but not ‘severe’. Our sample only contained 53 participants with ‘severe’ paruresis and was only able to detect large effects. Further research employing large sample sizes are needed to explore these factors to determine whether they have smaller effects.

Our findings indicate that paruresis may be much more prevalent in the general population than indicated by previous research. Our prevalence rates, however, were based only on the self-reported total SBS score which may have overestimated prevalence compared with other studies were formal clinical assessments were used for diagnosis. Although the diagnosis was self-reported, the responses to the SBS clearly indicate that a high proportion of individuals are struggling to manage with shy bladder syndrome symptoms. There are clear links with other anxiety disorders and self-esteem. Whether paruresis results in the anxiety disorders and low self-esteem or vice versa needs to be explored in more detail, possibly through in-depth qualitative work. More work is also needed to explore other factors that may have an influence on the development of paruresis. These include younger age groups, more diverse ethnic groups, education levels, family backgrounds and occupation. Substance abuse has been identified as playing a role in patients with overactive bladder, so it would also be interesting to determine whether this has any impact on paruresis development.^34^

This work has important implications for all organisations in relation to layout of buildings and support for staff. Organisations should consider building single gender, single occupancy toilets and locating toilets in quiet non-communal areas to limit anxiety associated with shy bladder syndrome. Considering music and running water in toilets may also help to overcome anxieties relating to micturition where individuals are conscious about others hearing them. Organisations should also consider providing resources such as counselling, mindfulness, information leaflets and self-help guidance to direct and to help individuals manage paruresis, as well as other anxiety conditions.

## supplementary material

10.1136/bmjopen-2024-086097 online supplemental file 1

10.1136/bmjopen-2024-086097 online supplemental file 2

## Data Availability

Data are available upon reasonable request.
