# Supplementary material for: Exploring paruresis (‘shy bladder syndrome’) and factors that may contribute to it: a cross-sectional UK survey study
Source: BMJ Open. 2024 Nov 17;14(11):e086097. doi: 10.1136/bmjopen-2024-086097 (PMC11574405; doi:10.1136/bmjopen-2024-086097)

a)Distribution of Total SBS scores

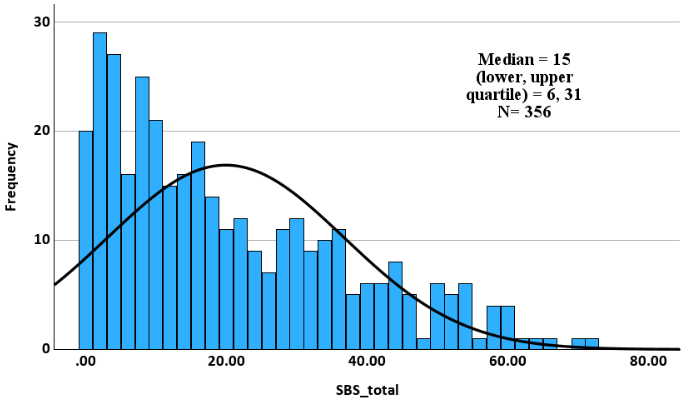

b)Distribution of RSES scores

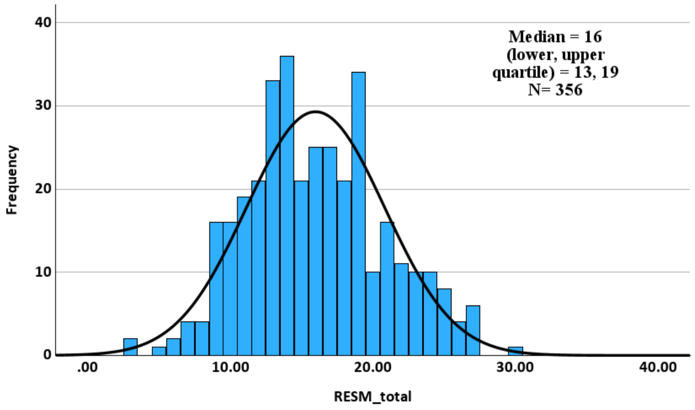

c)Distribution of School toilet experience scores

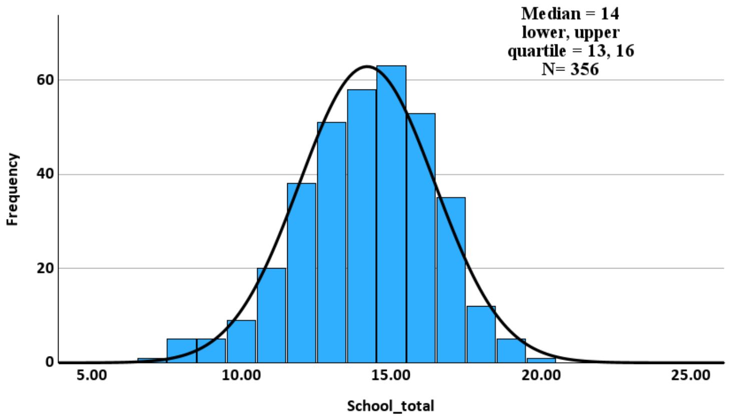

Supplement: online supplemental file 1 [file bmjopen-14-11-s001.pdf]
