# Supplementary material for: Exploring paruresis (‘shy bladder syndrome’) and factors that may contribute to it: a cross-sectional UK survey study
Source: BMJ Open. 2024 Nov 17;14(11):e086097. doi: 10.1136/bmjopen-2024-086097 (PMC11574405; doi:10.1136/bmjopen-2024-086097)
Supplement: online supplemental file 2 [file bmjopen-14-11-s002.pdf]

Supplementary information Table S1. Frequency of anxiety disorders in the 356 respondents.

Anxiety disorder	Frequency n (%)
**General anxiety disorder (GAD)**<br>*The main symptom of GAD is excessive worrying about different activities and events. You may feel anxious a lot of the time if you have GAD. You might feel 'on-edge' and hyper-alert to your surroundings.*	
Self-Diagnosed<br>Medically Diagnosed<br>No	84 (23.6%)<br>89 (25.0%)<br>183 (51.4%)
**Panic Disorder**<br>*Panic disorder means you have regular panic attacks with no particular trigger.*	
Self-Diagnosed<br>Medically Diagnosed<br>No	33 (9.3%)<br>31 (8.7%)<br>292 (82.0%)
**Social anxiety disorder**<br>*Social phobia is when you will have an intense fear or dread of social situations.*	
Self-Diagnosed<br>Medically Diagnosed<br>No	82 (23.0%)<br>45 (12.6%)<br>229 (64.3%)
**Health anxiety**<br>*Health anxiety is the constant worry that you are ill, or that you are going to get ill.*	
Self-Diagnosed<br>Medically Diagnosed<br>No	53 (14.9%)<br>17 (4.8%)<br>286 (80.3%)
**Phobia**<br>*A phobia is an overwhelming fear of an object, place, situation, feeling or animal.*	
Self-Diagnosed<br>Medically Diagnosed<br>No	91 (25.6%)<br>15 (4.2%)<br>250 (70.2%)
**Agoraphobia**<br>*Agoraphobia is a fear of being in situations where escape may be difficult.*	
Self-Diagnosed<br>Medically Diagnosed<br>No	50 (14.0%)<br>7 (2.0%)<br>299 (84.0%)
**Obsessive Compulsive Disorder (OCD)**<br>*You will have obsessions and/or compulsions if you have OCD. An obsession is a repeated unwelcome thought or image. These can be difficult to ignore. A compulsion is something you think about or do repeatedly to help relieve anxiety.*	
Self-Diagnosed<br>Medically Diagnosed<br>No	45 (12.6%)<br>29 (8.1%)<br>282 (79.2%)
**Post-traumatic stress disorder (PTSD)**<br>*PTSD is caused by a threatening situation, such as a car crash or abuse. You can feel anxious for months or years after the event, even if you weren't physically harmed at the time.*	
Self-Diagnosed<br>Medically Diagnosed<br>No	45 (12.6%)<br>34 (9.6%)<br>277 (77.8%)

**Skin picking**<br>*Skin picking is an impulse control disorder where you regularly pick at your skin and find it difficult to stop yourself from doing it.*	
Self-Diagnosed<br>Medically Diagnosed<br>No	80 (22.5%)<br>19 (5.3%)<br>257 (72.2%)
**Hair pulling**<br>*Hair pulling is an impulse control disorder where you feel the urge to pull out your hair and find it difficult to stop yourself from doing it.*	
Self-Diagnosed<br>Medically Diagnosed<br>No	40 (11.2%)<br>8 (2.2%)<br>308 (86.5%)

Supplementary information Table S2

Goodness of fit statistics for multivariable logistic models: a) Paruresis (Y) based on a total SBS score of 31 or more; b) Paruresis (Y) based on a total SBS score of 40 or more.

a) 'mild' paruresis (SBS total score 31 or more)

Omnibus Tests of Model Coefficients

		Chi-square	df	Sig.
Step 1	Step	44.817	9	<.001
	Block	44.817	9	<.001
	Model	44.817	9	<.001

Significance <0.05 indicating that our model (with our set variables as predictors) is better than the baseline model and that our model fit was appropriate.

Hosmer and Lemeshow Test

Step	Chi-square	df	Sig.
1	8.006	8	.433

Significance >0.05 supports good model fit.

Model Summary

Step	-2 Log likelihood	Cox & Snell R Square	Nagelkerke R Square
1	362.024[a]	.118	.174

a. Estimation terminated at iteration number 5 because parameter estimates changed by less than .001.

b) 'severe' paruresis (SBS total score 40 or more)

Omnibus Tests of Model Coefficients

		Chi-square	df	Sig.
Step 1	Step	24.820	9	.003

	**Block**	**24.820**	**9**	**.003**
	**Model**	**24.820**	**9**	**.003**

Significance <0.05 indicating that our model (with our set variables as predictors) is better than the baseline model and that our model fit was appropriate.

Hosmer and Lemeshow Test

**Step**	**Chi-square**	**df**	**Sig.**
**1**	**8.770**	**8**	**.362**

Significance >0.05 supports good model fit.

Model Summary

**Step**	**-2 Log likelihood**	**Cox & Snell R Square**	**Nagelkerke R Square**
**1**	**274.758[a]**	**.067**	**.118**

a. Estimation terminated at iteration number 5 because parameter estimates changed by less than .001.
