# Supplementary material for: Exploring paruresis (‘shy bladder syndrome’) and factors that may contribute to it: a cross-sectional UK survey study
Source: BMJ Open. 2024 Nov 17;14(11):e086097. doi: 10.1136/bmjopen-2024-086097 (PMC11574405; doi:10.1136/bmjopen-2024-086097)
Supplement: Supplementary file 3 [file bmjopen-2024-086097.reviewer_comments.pdf]

PEER REVIEW HISTORY

BMJ Open publishes all reviews undertaken for accepted manuscripts. Reviewers are asked to complete a checklist review form and are provided with free text boxes to elaborate on their assessment. These free text comments are reproduced below.

ARTICLE DETAILS

Title (Provisional)

Exploring Paruresis (‘Shy Bladder Syndrome’) and factors that may contribute to it. A cross-sectional United Kingdom survey study

Authors

Hutchings, Hayley Anne; Kehinde, Abi

VERSION 1 - REVIEW

**Reviewer**	**1**
**Name**	**Xu, Chang**
**Affiliation**	**Sichuan University, The Chinese Evidence-based Medicine Center & The Chinese Cochrane Center, West China Hospital**
**Date**	**06-Apr-2024**
**COI**	**none**

This is an interesting cross-sectional survey on a special topic, the Shy Bladder Syndrome. While it is well-conducted, I have several major concerns.

In this survey, the prevalance of Paruresis is as high as 67.1%. Althrough the authors explained this in the discussion, I think the 'sample difference'&'self-reported diagnose' is insufficient to explain the high prevalance. I would like to see more reasonable explaination.

Second, the authors used 'self-reported diagnose' method in the survey. The accuracy was not well-justified by a pilot study. They may want to use a small sample to measure the condition by questionairs and gold diagnose standard to show their method is accurcate enough.

Third, the author claimed their study is a large sample size study. While to my understanding in epidemiological studyies, a sample size of 300 to 400 is not so large for a cross-sectional study. Maybe the authors could show us the sample size of similar studies?

Fourth, the regression analysis was not well described. Can the authors present the distribution of the RSES score? How they model the regression if the RSES is not normally distributed? In addition, they independent variables were choose totally by experience, not

based on more reasonable methods such as the DAGs plot. So, I would like to ask the authors to present more details on statistical analysis.

Lastly, I feel the 'story' of current study is not large enough to BMJ Open. So, the authors may want to find some interesting results to make it more attractive and plentiful.

Reviewer **2**

Name **Wang, Xiao-Long**

Affiliation **Temple University, Department of Urology**

Date **21-Apr-2024**

COI **I agree with the review terms and conditions.I have no competing interests to disclose about the authors or the content of this manuscript.**

Thank you for the opportunity to review this manuscript titled "What is the prevalence of Paruresis (‘Shy Bladder Syndrome’) and are there any underlying factors that contribute to it? A cross-sectional survey study".

Major Comments:

1. The authors have raised an important topic that deserves more attention in the public domain. I appreciate their efforts in conducting this study and contributing to the literature on paruresis.

2. Statistical Analysis: Could the authors list the specific software packages used for statistical analysis instead of mentioning only the tests applied? It would be helpful to have a separate section or list detailing the statistical analyses performed and the software package used.

3. Participant Characteristics: The authors have provided demographic information on age, ethnicity, and gender. However, it would be valuable to include additional participant characteristics such as education level, family background, or occupation. These factors may play a role in understanding the participants' responses and the prevalence of paruresis.

4. Use of the term "significant": The authors have used the term "significant" throughout the manuscript. As mentioned in the comments, this term can be ambiguous, as it can mean "important" or "relevant" in plain English, but in a statistical context, it refers to a calculated p-value smaller than the prespecified statistical alpha. To avoid ambiguity, it is recommended to replace "significant" with the specific p-value or clarify the intended meaning in the context.

5. Substance Abuse: The high prevalence of self-reported paruresis in this study is surprising. It would be interesting to explore whether substance abuse could be a contributing factor, as

it is common in patients with overactive bladder (OAB). The authors could consider discussing this point in the Discussion section.

Additional Questions:

6. Could the authors provide more details on the sampling method used and the characteristics of the study population? This information would help assess the generalizability of the findings.

7. How did the authors handle missing data, if any, in the questionnaires and scales used in the study?

8. Were any measures taken to address potential biases, such as recall bias or social desirability bias, which could affect the self-reported responses?

Thank you again for the opportunity to review this manuscript.

VERSION 1 - AUTHOR RESPONSE

Reviewer: 1
Dr. Chang Xu, Sichuan University
Comments to the Author:
This is an interesting cross-sectional survey on a special topic, the Shy Bladder Syndrome. While it is well-conducted, I have several major concerns.
In this survey, the prevalence of Paruresis is as high as 67.1%. Although the authors explained this in the discussion, I think the 'sample difference '&'self-reported diagnose' is insufficient to explain the high prevalence. I would like to see more reasonable explanation.
Response: Thank you for these comments. We have now undertaken further analysis to the paper, defining both mild and severe paruresis. We have reported the prevalence rates for both these groups.

Second, the authors used 'self-reported diagnose' method in the survey. The accuracy was not well-justified by a pilot study. They may want to use a small sample to measure the condition by questionnaire and gold diagnose standard to show their method is accurate enough.
Response: Thank you for this feedback. We have added more detail to the manuscript to explain this in more detail. The basis for our cut-offs was based on the original SBS validation work. We have added more detail to explain this in the paper. The reviewer is correct in that the reporting of symptoms were based on self-report. Whilst we recognise that this has its limitations, it was beyond the scope of this study to compare this with a clinical diagnosis.

Third, the author claimed their study is a large sample size study. While to my understanding in epidemiological studies, a sample size of 300 to 400 is not so large for a cross-sectional study. Maybe the authors could show us the sample size of similar studies?

Response: We appreciate that our sample size may not be large in comparison with other cross-sectional studies. We have now removed this statement from the paper.

Fourth, the regression analysis was not well described. Can the authors present the distribution of the RSES score? How they model the regression if the RSES is not normally distributed? In addition, they independent variables were choose totally by experience, not based on more reasonable methods such as the DAGs plot. So, I would like to ask the authors to present more details on statistical analysis.
Response: Thank you for these helpful comments and we apologise for the lack of detail regarding the regression analysis. We have included an additional figure to the paper which presents the distribution of Shy Bladder Scale (SBS), Rosenberg Self-Esteem Scale (RSES) Score and School Toilet Experience scores. We have consulted with our statistician who has confirmed that normality is not a requirement for logistic regression. The selection of independent variables was based: 1) on existing literature proposing links between Shy Bladder Syndrome and gender, self-esteem and pre-existing anxiety disorders; and 2) initial exploration of the data using bivariate correlation (SBS Vs RSES, and SBS Vs were School Toilet Experience). Only those correlations that were significant were added to the logistic regression model. We have now added further detail to the paper.

Lastly, I feel the 'story' of current study is not large enough to BMJ Open. So, the authors may want to find some interesting results to make it more attractive and plentiful.
Response: We feel the paper is worthy of publication and represents one of the largest studies to date in this very limited field of research which deserves more public attention.

Reviewer: 2
Dr. Xiao-Long Wang, Temple University, Wuhan University
Comments to the Author:
Thank you for the opportunity to review this manuscript titled "What is the prevalence of Paruresis ('Shy Bladder Syndrome') and are there any underlying factors that contribute to it? A cross-sectional survey study".

Major Comments:

1. The authors have raised an important topic that deserves more attention in the public domain. I appreciate their efforts in conducting this study and contributing to the literature on paruresis.
Response: We thank the reviewer for their positive responses and agree that this is an under researched area that warrants increased research.

2. Statistical Analysis: Could the authors list the specific software packages used for statistical analysis instead of mentioning only the tests applied? It would be helpful to have a separate section or list detailing the statistical analyses performed and the software package used.
Response: The statistical package used is documented on page 7 under the Statistical analysis section along with the specific tests undertaken.

3. Participant Characteristics: The authors have provided demographic information on age, ethnicity, and gender. However, it would be valuable to include additional participant characteristics such as education level, family background, or occupation. These factors may play a role in understanding the participants' responses and the prevalence of paruresis.
Response: Thank you for these helpful comments. We agree that it would be valuable to explore these factors and their impact on paruresis. We were keen to make the survey anonymised and to limit the time to complete and hence the burden on participants. We therefore made a conscious decision to limit the data collected to reduce risk of identification as the participants were likely to be colleagues and students from the same institution as the authors. We have added to our discussion section that exploration of additional factors would be useful to explore in a future study.

4. Use of the term "significant": The authors have used the term "significant" throughout the manuscript. As mentioned in the comments, this term can be ambiguous, as it can mean "important" or "relevant" in plain English, but in a statistical context, it refers to a calculated p-value smaller than the prespecified statistical alpha. To avoid ambiguity, it is recommended to replace "significant" with the specific p-value or clarify the intended meaning in the context.
Response: Thank you for pointing this out. We have now removed the term significant where it does not relate to a significant difference. We have added statistically significant and the p-value, where this relates to the statistical analyses.

5. Substance Abuse: The high prevalence of self-reported paruresis in this study is surprising. It would be interesting to explore whether substance abuse could be a contributing factor, as it is common in patients with overactive bladder (OAB). The authors could consider discussing this point in the Discussion section.
Response: Thank you for this helpful comment. We have now added this point to the discussion section.

Additional Questions:

6. Could the authors provide more details on the sampling method used and the characteristics of the study population? This information would help assess the generalizability of the findings.
Response: All the demographic information we collected is presented in table 1. As noted in the response to Q3 from reviewer 2, we wanted to keep the survey as short as possible and avoid overburdening the participants. As the survey was sent to staff and students from the same institution as the authors, we wanted to avoid identification by collecting too much demographic information that could risk identifying participants. We therefore only collected demographic information which we deemed would be useful in determining factors that could influence paruresis development based on the literature. In addition, the response options were also grouped to avoid identification. We have added information to the paper. Details regarding sample are presented in the first paragraph of the methods section (pages 5 and 6).

7. How did the authors handle missing data, if any, in the questionnaires and scales used in the study?
Response: The survey was set up such that individual question responses to the SBS, RSES and school toilet experience questionnaires were mandatory. As such we did not need to undertake any imputation of the data. This information is presented on pages 6 and 7 of the paper.

8. Were any measures taken to address potential biases, such as recall bias or social desirability bias, which could affect the self-reported responses?
Response: We recognise these sources of bias may have influenced the responses given by the participants. However, as mentioned in the responses to Q6 and Q3 from reviewer 2, the participants were informed that all responses would be anonymised and that only the research team would have access to the data. Participants were free to withdraw from the study at any time and the limited demographic information collected mitigated any possible risk of identification of any participants. All staff and students from the institution were sent an email about the study but the survey was in no way linked to participants email addresses. We therefore believe we have taken appropriate measures to limit these biases.

VERSION 2 - REVIEW

**Reviewer**	**2**
**Name**	**Wang, Xiao-Long**
**Affiliation**	**Temple University, Department of Urology**
**Date**	**29-Jul-2024**

COI **I declare that I have no potentially conflicting or competing interests related to the review of this manuscript. This includes any personal, financial, intellectual, professional, political, or religious interests that could influence my assessment. I am committed to providing an unbiased and objective review based solely on the scientific merit of the work. I understand the importance of transparency in the open peer review process used by BMJ Open and confirm that I can review this manuscript without any conflicts that would compromise the integrity of the review process.**

Thank you for addressing my comments satisfactorily. Your clarifications and additions have improved the manuscript. This study on paruresis addresses an important, under-researched topic in the LUTS study. Given its significance and quality, I believe this article would greatly

interest BMJ Open's readership, contributing valuable insights and potentially stimulating further research in this area.

**Reviewer**	**3**
**Name**	**Scandrett, Katie**
**Affiliation**	**University of Birmingham**
**Date**	**30-Aug-2024**
**COI**	**None**

Thank you for the opportunity to review this well-written and interesting submission. Whilst I think the findings from this study are useful and worth publishing, I think parts of the analysis need to be revised. In particular, I am unsure whether a SBS score of >9 is a suitable threshold for defining mild paruresis based on the included reference, and I also think some of the t-tests performed are inappropriate. Please see my comments below.

Major comments

Why was a score of less than 9 chosen for mild paruresis when the mean score for those without paruresis is 9.2? I can't find the range or interquartile range of SBS scores for the non-paruresis group in the Deacon 2012 paper, but the mean score was 9.2 in the group without paruresis, and the SD was 13.6. Therefore, I question whether a score of >9 is a suitable threshold for defining 'mild' paruresis. It also seems unlikely that 67% of the sample have mild paruresis given that previous literature has estimated the prevalence to be between 2.8% and 16.4%.

Is there any evidence to support the cut-offs chosen for average and low self-esteem as measured by the RSES?

Is there any evidence to support the cut-offs chosen for negative and adequate/positive experience as measured by the school toilet experience questionnaire?

How was a 'small to medium effect size' defined in the sample size calculation? Please include more detail about how the sample size was determined. Also, only 53 participants had severe paruresis, so the minimum sample size requirement (n=90) has not been reached for this outcome.

The following sentence is unclear: 'We explored the relationship between existing society anxiety disorders and SBS scores using independent sample t test where the presence of any social anxiety disorder was compared to having no social anxiety disorders.'. Are the authors referring to comparison of mean SBS scores in those with one or more social anxiety disorder compared to those with no social anxiety disorders? Also, was the normality of the SBS score checked before the t-test was conducted? Is the assumption that the variance is approximately equal in both groups appropriate?

From the histograms in Figure 1, it is clear that the distribution of the total SBS score is skewed. Therefore, I question whether it is appropriate to conduct t-tests using mean SBS scores.

In Table 3, the authors present t-tests for paruresis versus no paruresis groups. The paruresis groups are defined by the score on the SBS. The mean SBS score is therefore going to be different in these two groups by design, since these groups were defined using a cut-off for the SBS. Results presented in Table 4 however, are more informative since the presence or absence of anxiety disorders was not defined by the SBS, RSES or the school toilet experience scale. Also, description of the t-tests conducted is unclear, as mentioned in my previous comment.

In the results section it is stated that 'Higher SBS scores (i.e. worse paruresis) were linked to a lower RSES score (i.e. lower self-esteem) for the total SBS score and all SBS sub-scales.'. However, the correlation coefficient for SBS interference vs RSES 0.18 is positive.

Results from the logistic regression model have not been presented in enough detail. This analysis is more informative than the correlation coefficients but is discussed less. However, I question whether the logistic regression model for mild paruresis is appropriate given my previous comment regarding the threshold of 9 to define mild paruresis. Please include the odds ratios and 95% CIs when discussing the results from this analysis. For example: 'the odds of having severe paruresis were higher for males than females (OR 2.58, 95% CI: 1.31-5.0), after adjusting for the other covariates included in the model'. Also, please note that risk is not the same as odds, and since this is a logistic regression model, results must be interpreted accordingly.

Why are p-values given for the reference categories (i.e. aged 18-30, female)? P-values should not be given for the reference category and the statistical software should not generate these. Perhaps these p-values are from a likelihood ratio test?

The categories for 60-70 and over 70 need to be combined in the logistic regression model since there was only one participant aged over 70. This likely why the odds ratio is so large with an inestimable CI.

Since the minimum sample size requirement was not met for the severe paruresis outcome, as mentioned in my previous comment, did the authors consider using a modelling technique for a continuous outcome to avoid dichotomisation of the SBS scale?

I question the threshold of <0.05 to determine statistical significance is appropriate, especially given the large number of statistical tests performed. Did the authors consider an adjustment for multiple testing (for example the Bonferroni correction)? Also, please report the actual p-values rather than p>0.05 or p<0.05. A p-value of 0.0001 has a very different interpretation to a p-value of 0.049.

The limitations of the study need to be expanded on in the discussion.

Minor comments

A brief sentence to explain how the prevalence of mild and severe paruresis were determined in the 'Design' section of the abstract may be beneficial.

Methods used to analyse the data from the questionnaire are not mentioned in the abstract.

Please include the effect measure estimates (i.e. the odds ratios) that demonstrate the significant predictors of paruresis in the abstract. Stating that something is statistically significant predictor does not demonstrate the magnitude of the association.

Perhaps some of the paragraphs in the introduction could be combined so that it reads better.

Please use 'percentages' rather '%' on line 16 page 8.

Did the authors consider presenting medians and inter-quartile ranges for non-normally distributed scores (i.e. SBS score)?

Please include in the methods section that age was collected as a categorical variable.

Why was the non-parametric Spearman correlation chosen to assess correlations between the scores?

Please include in the methods that a multivariable logistic regression model was used.

How was goodness of fit of the logistic regression model assessed?

Please improve the presentation of the figures showing the distribution of the SBS, RSES and school toilet experience scores. I would reconsider inclusion of these figures in the main text.

Table 1, Table 2 and Supplementary Table 1 could be combined. The number of people with 1 or more, and no anxiety disorders should also be included. I would recommend presenting the median and interquartile range for non-normally distributed variables.

The section reporting the correlation coefficients is quite hard to read. Perhaps the results could be presented in a table?

The log odds ratios and the standard errors do not need to be presented in Table 5.

VERSION 2 - AUTHOR RESPONSE

Reviewer: 2
Dr. Xiao-Long Wang, Temple University, Wuhan University
Comments to the Author:
Thank you for addressing my comments satisfactorily. Your clarifications and additions have improved the manuscript. This study on paruresis addresses an important, under-researched topic in the LUTS study. Given its significance and quality, I believe this article would greatly interest BMJ Open's readership, contributing valuable insights and potentially stimulating further research in this area.

Response: We thank reviewer 2 for their positive evaluation.

Reviewer: 3
Miss Katie Scandrett, University of Birmingham
Comments to the Author:
Thank you for the opportunity to review this well-written and interesting submission. Whilst I think the findings from this study are useful and worth publishing, I think parts of the analysis need to be revised. In particular, I am unsure whether a SBS score of >9 is a suitable threshold for defining mild paruresis based on the included reference, and I also think some of the t-tests performed are inappropriate. Please see my comments below.

Major comments

Why was a score of less than 9 chosen for mild paruresis when the mean score for those without paruresis is 9.2? I can't find the range or interquartile range of SBS scores for the non-paruresis group in the Deacon 2012 paper, but the mean score was 9.2 in the group without paruresis, and the SD was 13.6. Therefore, I question whether a score of >9 is a suitable threshold for defining 'mild' paruresis. It also seems unlikely that 67% of the sample have mild paruresis given that previous literature has estimated the prevalence to be between 2.8% and 16.4%.

Response: Thank you for these helpful comments. The original cut-off for defining 'mild paruresis' was based on of a SBS of greater than 9 (which was based on the original Deacon paper developing the SBS where the mean total SBS scores for control non-paruresis individuals was 9.20). We appreciate however that this cut-off might be too low for defining clinical paruresis and estimating prevalence. We have now reanalysed the data using cut-off values of greater than or equal to 31 to define 'mild' paruresis in addition to our cut-off of 40 or more to define 'severe' paruresis. These thresholds were used in the Deacon paper to assess the sensitivity and specificity of the SBS against a clinical assessment score for paruresis. We appreciate that these cut-offs may still not be comparable to clinical paruresis prevalence and have discussed this also in the paper.

Is there any evidence to support the cut-offs chosen for average and low self-esteem as measured by the RSES?

Response: These cut-offs are based on definitions from existing literature. We have now cited an appropriate reference alongside these cut-offs.

Is there any evidence to support the cut-offs chosen for negative and adequate/positive experience as measured by the school toilet experience questionnaire?

Response: As this questionnaire was adapted from a previous questionnaire which has no defined cut-offs, we defined a negative school toilet experience score based on individuals scoring negatively to more than 50% of questions, i.e. having a score of 10 or less. This information has been added to the paper to further clarify.

How was a 'small to medium effect size' defined in the sample size calculation? Please include more detail about how the sample size was determined. Also, only 53 participants had severe

paruresis, so the minimum sample size requirement (n=90) has not been reached for this outcome.

Response: We have now added more detail in the sample size section based on sample size guidance for logistic regression. We apologise that the original sample size calculation was incorrect and based on six predictors, whereas our model only used five. The sample size requirement was between 43 and 92 participants. Our redefined cut-off of 31 for 'mild' and 40 for 'severe' paruresis have reached the required minimum suggested sample sizes for a medium and large effect respectively.

The following sentence is unclear: 'We explored the relationship between existing society anxiety disorders and SBS scores using independent sample t test where the presence of any social anxiety disorder was compared to having no social anxiety disorders.'. Are the authors referring to comparison of mean SBS scores in those with one or more social anxiety disorder compared to those with no social anxiety disorders? Also, was the normality of the SBS score checked before the t-test was conducted? Is the assumption that the variance is approximately equal in both groups appropriate?

Response: We apologise for the confusing language. We have now changed this is the paper. We explored SBS scores in those individuals with and without a social anxiety disorder. We have explored the normality of the data using the Kolmogorov-Smirnov Test and can confirm that the SBS, RSES and school toilet experience score data were not normally distributed. We have now used the non-parametric tests to compare scores within these groups. We have amended the statistical analysis section to reflect these changes.

From the histograms in Figure 1, it is clear that the distribution of the total SBS score is skewed. Therefore, I question whether it is appropriate to conduct t-tests using mean SBS scores.

Response: Thank you for this observation. We have now checked the normality of the data using Kolmogorov-Smirnov normality tests and can confirm that the data are not normally distributed. We have therefore used non-parametric Mann-Whitney U tests rather that independent samples t tests throughout and have amended the relevant sections in the paper. As data were not normally distributed we have now presented the data as median (interquartile range) rather than mean (SD).

In Table 3, the authors present t-tests for paruresis versus no paruresis groups. The paruresis groups are defined by the score on the SBS. The mean SBS score is therefore going to be different in these two groups by design, since these groups were defined using a cut-off for the SBS. Results presented in Table 4 however, are more informative since the presence or absence of anxiety disorders was not defined by the SBS, RSES or the school toilet experience scale. Also, description of the t-tests conducted is unclear, as mentioned in my previous comment.

Response: Thank you for these observations. We agree that the SBS score will be different by design in these two groups but still thought it would be useful to illustrate the range of scores within each of the groups. We have not statistically analysed the SBS scores across the groups based on the reviewers comments and have removed significance tests values. We have now reanalysed our data based on the 'mild' and 'severe' SBS cut-offs of 31 and 40 respectively and have amended Table 3 to reflect these. We now also present the findings as medians (IQR)

based on the fact that the data are not normally distributed ana analysis using the Mann-Whitney U test.

In the results section it is stated that 'Higher SBS scores (i.e. worse paruresis) were linked to a lower RSES score (i.e. lower self-esteem) for the total SBS score and all SBS sub-scales.'. However, the correlation coefficient for SBS interference vs RSES 0.18 is positive.

Response: Thank you for highlighting this. This is an error on our part and the figure should read -0.18. We have now corrected this in the manuscript.

Results from the logistic regression model have not been presented in enough detail. This analysis is more informative than the correlation coefficients but is discussed less. However, I question whether the logistic regression model for mild paruresis is appropriate given my previous comment regarding the threshold of 9 to define mild paruresis. Please include the odds ratios and 95% CIs when discussing the results from this analysis. For example: 'the odds of having severe paruresis were higher for males than females (OR 2.58, 95% CI: 1.31-5.0), after adjusting for the other covariates included in the model'. Also, please note that risk is not the same as odds, and since this is a logistic regression model, results must be interpreted accordingly.

Response: Thank you for these helpful comments. We have now presented the findings in more detail. We now present findings for 'mild' and 'severe' paruresis using the defined cut-offs of 31 and 40 from the original Deacon paper. We have included the odds ratios and 95% confidence intervals and changed the terminology used.

Why are p-values given for the reference categories (i.e. aged 18-30, female)? P-values should not be given for the reference category and the statistical software should not generate these. Perhaps these p-values are from a likelihood ratio test?

Response: We apologise for the confusion. We have now removed these p-values.

The categories for 60-70 and over 70 need to be combined in the logistic regression model since there was only one participant aged over 70. This likely why the odds ratio is so large with an inestimable CI.

Response: We have now combined these categories in the logistic regression model.

Since the minimum sample size requirement was not met for the severe paruresis outcome, as mentioned in my previous comment, did the authors consider using a modelling technique for a continuous outcome to avoid dichotomisation of the SBS scale?

Response: Thank you for these comments. In order to align the findings with existing studies we felt a logistic regression model would be a better approach. We do however recognise the limitations of this approach especially given the numbers are limited in the 'severe' group. We have now adopted the cut-offs of 31 and 40 specified in the original Deacon paper as our cut-offs for defining paruresis. We have amended information relating to the sample size as the number of risk factors used was in fact 5 and not 6. We have also discussed as a limitation that the sample size was only able to detect large effect sizes in the 'severe' paruresis group.

I question the threshold of <0.05 to determine statistical significance is appropriate, especially given the large number of statistical tests performed. Did the authors consider an adjustment for multiple testing (for example the Bonferroni correction)? Also, please report the actual p-values rather than p>0.05 or p<0.05. A p-value of 0.0001 has a very different interpretation to a p-value of 0.049.

Response: Thank you for this feedback. We have removed statistical analysis comparing paruresis and non-paruresis groups as discussed above. We have therefore now only performed a limited number of analyses based on a priori hypotheses. Even with Bonferroni adjustments these analyses would still be significant. We have therefore not reported these.

We have now reported p values larger than 0.01 should to two decimal places, and those between 0.01 and 0.001 to three decimal places; p values smaller than 0.001 are reported as p<0.001[1].

1. Aguinis H, Vassar M, Wayant C. On reporting and interpreting statistical significance and p values in medical research. BMJ Evid Based Med. 2021;26(2):39-42. Epub 20191115. doi: 10.1136/bmjebm-2019-111264. PubMed PMID: 31732498; PubMed Central PMCID: PMCPMC8005799.

The limitations of the study need to be expanded on in the discussion.

Responses: We have now expanded the limitations section of the discussion.

Minor comments

A brief sentence to explain how the prevalence of mild and severe paruresis were determined in the ‘Design’ section of the abstract may be beneficial.

Response: Thank you. We have now added details regarding how prevalence were defined in the abstract.

Methods used to analyse the data from the questionnaire are not mentioned in the abstract.

Response: Thank you. We have now added how data were analysed to the abstract.

Please include the effect measure estimates (i.e. the odds ratios) that demonstrate the significant predictors of paruresis in the abstract. Stating that something is statistically significant predictor does not demonstrate the magnitude of the association.

Response: *Odds ratios have now been added to the abstract.*

Perhaps some of the paragraphs in the introduction could be combined so that it reads better.

Response: We have now combined some paragraphs in the introduction to improve readability.

Please use ‘percentages’ rather ‘%’ on line 16 page 8.

Response: We have now used percentages rather than %.

Did the authors consider presenting medians and inter-quartile ranges for non-normally distributed scores (i.e. SBS score)?

Response: We have now explored the data for normality and present median (interquartile range) for those data that are nor normally distributed.

Please include in the methods section that age was collected as a categorical variable.

Response: We have now added that age was collected as a categorical variable to the methods section.

Why was the non-parametric Spearman correlation chosen to assess correlations between the scores?

Response: We have explored the data and as the variables were not normally distributed we used the non-parametric Spearman test.

Please include in the methods that a multivariable logistic regression model was used.

Response: We have added that a multivariable logistic regression model was used to the methods.

How was goodness of fit of the logistic regression model assessed?

Response: We have provided details of how the goodness of fit of the regression model was assessed. We have also included the results of the goodness of fit assessment in the supplementary materials.

Please improve the presentation of the figures showing the distribution of the SBS, RSES and school toilet experience scores. I would reconsider inclusion of these figures in the main text.

Response: We have now amended the figures to improve the presentation. It is our intention to include the figure within the text. The BMJ Open submission requirements are however that figures are submitted as a separate upload.

Table 1, Table 2 and Supplementary Table 1 could be combined. The number of people with 1 or more, and no anxiety disorders should also be included. I would recommend presenting the median and interquartile range for non-normally distributed variables.

Response: Thank you for these helpful suggestions. We have now combined Tables 1, 2 and Supplementary Table 1 and included no or one or more anxiety disorders. We now present the data as median (interquartile range).

The section reporting the correlation coefficients is quite hard to read. Perhaps the results could be presented in a table?

Response: We have now removed most of the text and present this information in a table.

The log odds ratios and the standard errors do not need to be presented in Table 5.

Response: Thank you. We have now removed these from Table 5.

Reviewer: 2
Competing interests of Reviewer: I declare that I have no potentially conflicting or competing interests related to the review of this manuscript. This includes any personal, financial, intellectual, professional, political, or religious interests that could influence my assessment. I am committed to providing an unbiased and objective review based solely on the scientific merit of the work. I understand the importance of transparency in the open peer review process used by BMJ Open and confirm that I can review this manuscript without any conflicts that would compromise the integrity of the review process.

Reviewer: 3
Competing interests of Reviewer: None

VERSION 3 - REVIEW

**Reviewer**	**3**
**Name**	**Scandrett, Katie**
**Affiliation**	**University of Birmingham**
**Date**	**07-Oct-2024**
**COI**	

I would like to thank the authors for their comprehensive responses to my previous comments and for updating the manuscript accordingly. I think the manuscript is much improved as a result. However, I still have a few outstanding queries. Please see my comments below.

Perhaps results from the logistic regression models should be focussed on in the abstract. These results are most informative and would leave more words available for the conclusion section.

The Kolmogorov-Smirnov test for normality is not commonly used. Instead, normality is usually determined by visually inspecting the distribution of a variable.

The measures to assess goodness of fit have not been included in the methods. Which models is the Omnibus test comparing? Was the baseline model compared to the model with all covariates included? Did the goodness of fit measures suggest that the model fit was appropriate?

Were the covariates all selected based on previous literature? They are all mentioned in the introduction in the context of the potential link with paruresis. However following the correlation coefficient results, it is stated that the RSES and school toilet experience scores were ‘strong candidates’ for the logistic regression model. If the correlation coefficients were

not statistically significant, would these covariates still have been included in the logistic regression model? The method for selecting covariates for inclusion in the logistic regression models is unclear.

Cohen's d (which is what the effect measure criteria appear to be based on) relates to the standardised mean difference and R squared is not used for logistic regression. Therefore, I question whether same sample size calculation is appropriate.

Please also include the number of participants when using percentages.

Sorry if my previous comment was unclear, but I do not recommend including the histograms in the main text. Also, the highest score for the SBS was 71, but it does not logically follow that many participants were experiencing debilitating paruresis symptoms.

The IQR should be presented as (Lower quartile, Upper Quartile) rather than (Upper quartile – Lower quartile).

The text explaining the results in table 4 is quite hard to read. I think that the logistic regression model better investigates relationships between the various scales/covariates and paruresis whilst allowing for adjustment of other covariates. I would suggest reducing this text, and instead pointing the reader to table 4 if they wish to see correlation coefficient results.

The Mann-Whitney U test is not a test for the difference in medians. Instead, it tests whether there is a statistically significant difference in the ranking of values between two groups. If the distribution of the two groups is similar, it can be inferred that there is a difference between the two medians.

When interpreting odds ratios, it is better to avoid phrases such as 'XX times more likely' since this may be interpreted as risk rather than odds. For example, 'the odds of mild paruresis were higher for men than women (adjusted OR 3.39; 95% CI 1.90-6.06)'.

Please include that the odds ratios are adjusted when describing results from the logistic regression models.

What is the 'younger age category' (lines 58/59 page 12)?

VERSION 3 - AUTHOR RESPONSE

Reviewer: 3
Miss Katie Scandrett, University of Birmingham
Comments to the Author:
I would like to thank the authors for their comprehensive responses to my previous comments and for updating the manuscript accordingly. I think the manuscript is much improved as a result. However, I still have a few outstanding queries. Please see my comments below.

Response: We thank reviewer 3 for their positive response to our revised manuscript. We have responded to the outstanding queries below.

Perhaps results from the logistic regression models should be focussed on in the abstract. These results are most informative and would leave more words available for the conclusion section.

Response: We have now updated the abstract to focus more on the results from the logistic regression and added further information to the abstract conclusion.

The Kolmogorov-Smirnov test for normality is not commonly used. Instead, normality is usually determined by visually inspecting the distribution of a variable.

Response: Whilst we accept that a visual inspection of the data is useful for an initial assessment of normality, this approach is sometimes regarded as being unreliable and does not guarantee that the distribution is normal[1]. We have therefore also applied Kolmogorov-Smirnov and Shapiro-Wilk tests to confirm normality. We have added this information to the manuscript.

1. Ghasemi A, Zahediasl S. Normality tests for statistical analysis: a guide for non-statisticians. Int J Endocrinol Metab. 2012;10(2):486-9. Epub 20120420. doi: 10.5812/ijem.3505. PubMed PMID: 23843808; PubMed Central PMCID: PMCPMC3693611.

The measures to assess goodness of fit have not been included in the methods. Which models is the Omnibus test comparing? Was the baseline model compared to the model with all covariates included? Did the goodness of fit measures suggest that the model fit was appropriate?

Response: We have now included information regarding goodness of fit assessment in the methods. We have also added that the baseline model was compared to the model with all covariates included. We have highlighted in the supplementary information table 2 that the model fit was appropriate.

Were the covariates all selected based on previous literature? They are all mentioned in the introduction in the context of the potential link with paruresis. However following the correlation coefficient results, it is stated that the RSES and school toilet experience scores were 'strong candidates' for the logistic regression model. If the correlation coefficients were not statistically significant, would these covariates still have been included in the logistic regression model? The method for selecting covariates for inclusion in the logistic regression models is unclear.

Response: Covariates were initially identified from previous literature. We further confirmed possible relationships between paruresis and self-esteem and school toilet experience using bivariate correlations. Only those correlation coefficients that were statistically significant were included in the logistic regression models. We have added this detail to manuscript to clarify.

Cohen's d (which is what the effect measure criteria appear to be based on) relates to the standardised mean difference and R squared is not used for logistic regression. Therefore, I question whether same sample size calculation is appropriate.

Response: We apologise for the confusion regarding the sample size calculation. We have now consulted with a statistician who has directed us to more recent guidance regarding sample size calculation for logistic regression. We have amended the sample size calculation section as a result.

Please also include the number of participants when using percentages.

Response: We have now included the numbers of participants where we present percentages.

Sorry if my previous comment was unclear, but I do not recommend including the histograms in the main text. Also, the highest score for the SBS was 71, but it does not logically follow that many participants were experiencing debilitating paruresis symptoms.

Response: We have now removed figure 1 from the main text. We have however included it as a supplementary figure to illustrate that the data were not normally distributed. We have removed the statement implying that many participants were experiencing debilitating paruresis symptoms.

The IQR should be presented as (Lower quartile, Upper Quartile) rather than (Upper quartile – Lower quartile).

Response: We have now amended the manuscript and accompanying tables to present the results as median (lower quartile, upper quartile) rather than median (IQR).

The text explaining the results in table 4 is quite hard to read. I think that the logistic regression model better investigates relationships between the various scales/covariates and paruresis whilst allowing for adjustment of other covariates. I would suggest reducing this text, and instead pointing the reader to table 4 if they wish to see correlation coefficient results.

Response: Thank you. We have now reduced the text in this section and direct readers to table 4.

The Mann-Whitney U test is not a test for the difference in medians. Instead, it tests whether there is a statistically significant difference in the ranking of values between two groups. If the distribution of the two groups is similar, it can be inferred that there is a difference between the two medians.

Response: Thank you. We have now removed the word median when referring to the Mann-Whitney U test results.

When interpreting odds ratios, it is better to avoid phrases such as 'XX times more likely' since this may be interpreted as risk rather than odds. For example, 'the odds of mild paruresis were higher for men than women (adjusted OR 3.39; 95% CI 1.90-6.06)'.

Response: Thank you for this feedback. We have now adjusted the terminology throughout the manuscript that describes the odds ratio results from the logistic regression.

Please include that the odds ratios are adjusted when describing results from the logistic regression models.

Response: We have now added that the odds ratios are adjusted when describing the results from the logistic regression models.

What is the 'younger age category' (lines 58/59 page 12)?

Response: We have now added what we define as the younger age category (18-30 years) to the discussion.
